# The Intake of a Cafeteria Diet in Nursing Rats Alters the Breast Milk Concentration of Proteins Important for the Development of Offspring

**DOI:** 10.3390/nu12082470

**Published:** 2020-08-17

**Authors:** Catalina Amadora Pomar, Juana Sánchez, Andreu Palou

**Affiliations:** 1 Laboratory of Molecular Biology, Nutrition and Biotechnology (Nutrigenomics and Obesity), University of the Balearic Islands, 07122 Palma, Spain; c.pomar@uib.es (C.A.P.); andreu.palou@uib.es (A.P.); 2 Instituto de Investigación Sanitaria Illes Balears, 07010 Palma, Spain; 3 CIBER Fisiopatología de la Obesidad y Nutrición (CIBEROBN), Instituto de Salud Carlos III (ISCIII), 28029 Madrid, Spain

**Keywords:** milk composition, bioactive proteins, lactation

## Abstract

We aimed to analyse the effects of maternal intake of an unbalanced diet during lactation in the composition and the levels of proteins present in milk. Milk samples from control nursing dams (C-dams) or from nursing dams fed a cafeteria diet during lactation (CAF-dams) were obtained. We conducted a proteomic approach to identify significantly altered proteins in breast milk of C- and CAF-dams, and evaluated the levels of leptin, adiponectin and irisin for their implication in energy homeostasis. One-dimensional SDS-polyacrylamide gel electrophoresis (SDS-PAGE), followed by matrix-assisted laser desorption/ionization time-of-flight mass spectrometry (MALDI-TOF MS), revealed that the bands that presented a lower intensity in CAF-dams than control contain some caseins (α-S1-casein, α-S2-casein like B, and β-casein), α-lactalbumin and haptoglobin. Leptin and adiponectin levels were greater in the breast milk of CAF-dams than in controls, while levels of irisin were lower. In summary, the relative concentration of bioactive peptides was influenced by maternal diet consumption during lactation; these changes at early stages of life could influence the phenotypic traits of the offspring.

## 1. Introduction

Bioactive proteins in breast milk are likely to contribute to the advantages of breastfeeding through enzyme activities, enhancement of the nutrient absorption, modulation of the immune system and growth stimulation (reviewed in [1]). For example, lactoferrin, lysozyme, α-lactalbumin, and some caseins, such as β-casein and κ-casein, as well as immunoglobulins, are bioactive proteins present in human milk [1]. Caseins are a heterogeneous group of phosphoproteins that are included in micelles, together with other components such as calcium phosphate [2]. Thus, they are a source of phosphate and calcium for the mineralization process, and of amino acids in the offspring [2]. Breastfeeding is known to improve the development of the immune system in new-born babies [3]. In addition to its nutritional value, casein also plays an immunologic relevant role in the nurslings [4]. Another, principal protein present in milk is α-lactalbumin; it provides a well-balanced supply of essential amino acids to the growing infant. In addition, it plays a central biochemical role in the mammary gland as a regulatory subunit of lactose synthase. This enables lactose synthase to synthesize lactose, the major carbohydrate component of milk, thus facilitating milk production and secretion. Moreover, α-lactalbumin binds the essential divalent cations (calcium, zinc), and may facilitate the absorption of these minerals [5].

In addition, breastfeeding is suggested to protect against later obesity, which could partially be attributable to bioactive compounds present in breast milk, but absent in infant formula [6,7]. However, the specific components of breast milk responsible for these beneficial effects have not yet been identified. In this regard, leptin, adiponectin and irisin are known proteins with a role in the energy homeostasis, and they are also present in milk. Their levels in milk could be influenced by maternal status and/or diet during lactation. For example, obese mothers had higher milk leptin levels [8,9] and adiponectin [10] than normal weight mothers. In contrast, lactating women with gestational diabetes mellitus presented lower irisin concentrations in milk than healthy lactating women [11]. Leptin present in breast milk seems to be critical for the development of the neonate (revised in [12]). In animal models, leptin taken at physiological doses during the suckling period has an essential neurotrophic action and is an important signal for the development of hypothalamic circuits involved in food intake and body weight control, and confers a certain protection against body weight gain an insulin resistance in adulthood (revised in [12]). Given the role of adiponectin in the regulation of metabolism, the discovery of its presence in breast milk could influence the growth and adiposity of offspring. High levels of adiponectin in milk are associated with later higher body weight, body weight gain or adiposity in infants, thus, it may be a risk factor for childhood overweight [13,14,15]. Irisin levels in breast milk may contribute to the development of catch up growth of the offspring [16] and may be implicated in postnatal adaptation, with respect to thermoregulation, vascular adaptation, glucose metabolism, lung function and fluid homeostasis [17]. All in all, these studies reveal the importance of maternal nutrition and/or status in the levels of these proteins, but limited information has been reported on whether these specific milk proteins could have a key role in the metabolic programming in offspring.

Previously, we have described that total breast milk protein content decreases throughout lactation, and was lower in cafeteria diet-fed dams than in control dams, especially at the first days of lactation [18]. In view of the evidence suggesting the importance of specific compounds in milk and that limited information has been reported on whether specific milk proteins are influenced by maternal diet during lactation, we also considered it of interest to extend our investigation and to asses if changes in the quantity of specific proteins are capable of predisposing offspring to metabolic alterations later in life. Therefore, the aim of the present study was to investigate the changes in the amount of specific proteins in breast milk of dams fed with an obesogenic diet during lactation. For this purpose, we conducted an SDS-PAGE followed by matrix-assisted laser desorption/ionization time-of-flight mass spectrometry (MALDI-TOF MS) in milk of control and cafeteria fed dams, to screen for specific proteins altered by maternal diet, and also evaluated the concentration of leptin, adiponectin and irisin. We postulated that the intake of the maternal cafeteria diet affected not only total protein content in milk, but also milk specific proteins/peptides composition.

## 2. Materials and Methods

### 2.1. Ethics Statement

All animal experimental procedures followed in this study were reviewed and approved by the Bioethical Committee of the University of the Balearic Islands (Ref 3513 (26 March 2012)), and guidelines for the use and care of laboratory animals of the university were followed.

### 2.2. Animals and Experimental Design

**Figure 1:**
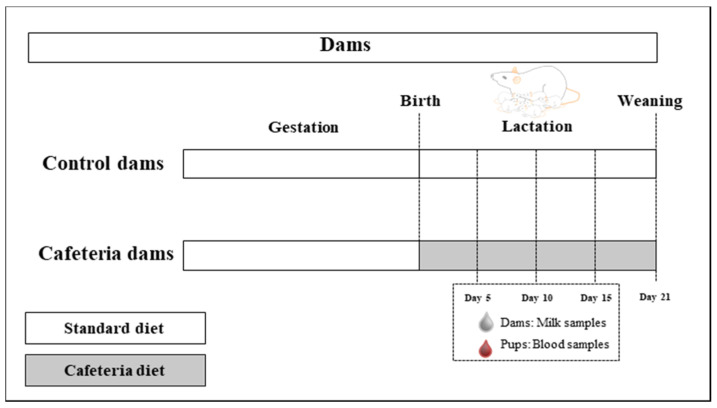
Representative scheme of the animal model used in this study.

The study was performed on dams and their breast milk from a previously published study conducted at the University of the Balearic Islands in 2013 [18]. Virgin dams fed with a standard chow diet (control) (3300 kcal kg^−1^; Panlab, Barcelona, Spain) were mated to control males. At postnatal day 1 (PN1), litters were adjusted to 10 pups per dam, and dams were assigned to either control or cafeteria group (*n* = 8) (Figure 1). During suckling period, control dams (C-dams) continued with the control diet, and dams of the cafeteria group (CAF-dams) were exposed to a cafeteria diet in addition to the standard chow. The cafeteria diet included: biscuits with a Majorcan sausage (‘sobrasada’) and with liver pâté, salted peanuts, chocolate, candies, carrots, fresh bacon, cheese, sugared milk (20% *w/v*) and a Majorcan pastry (‘ensaimada’) [18]. Due to the variety of highly palatable foods, the cafeteria diet has been shown to be an interesting tool to induce voluntary hyperphagia [19,20], and results in a rapid increase in body weight and alters the metabolic factors clustered in metabolic syndrome [21,22].

At three time points of lactation (days 5, 10 and 15), milk samples from dams and plasma of pups were collected. For milk extraction, dams were previously separated from their pups for 2–3 h. Then, 0.4 IU kg^−1^ of body weight of oxitocin (Facilpart, Laboratory syva s.a.u, León, España) was administered intraperitoneally to dams. After 5 min, dams were anesthetised using isoflurane (IsoFlo, Abbott Laboratories Ltd., North Chicago, IL, USA), and were kept anesthetised throughout the extraction procedure, to reduce their stress level. Milk was extracted manually from all teats, mixed and stored at −80 °C until further analysis. During the separation of the dams from the offspring, a sample of blood in a capillary was collected from the end of the tail of the pups. Plasma was obtained by centrifugation, and samples of the same litter were pooled and stored at −20 °C until further analysis. 

### 2.3. One-Dimensional SDS-Polyacrylamide Gel Electrophoresis

The electrophoretic separation of milk proteins on day 15 was carried under denaturing conditions in a 2% polyacrylamide stacking gel, and in a 15% polyacrylamide resolving gel. In gel, 5 μL of whole milk diluted 1/100 in phosphate buffered saline (PBS) with 10 μL of loading buffer was loaded. Previously, breast milk proteins were boiled for 3 min in laemmli sample buffer, containing 5% 2-beta-mercaptoethanol. Electrophoresis was carried out in tris-glycine, under the following conditions: 10 min at 100 V and left overnight at 80 V.

### 2.4. Staining and Quantification

Once the separation of proteins by molecular size was complete, the gel was fixed and stained with Coomassie blue. When the bands were well defined and the bottom of the gel well faded, the gel was scanned with the Chemi Genius Bio Imaging System (Syngene). The intensity of the stained bands was quantified using Gene Tools from Syngene, to screen for differences between CAF- and C-dams.

### 2.5. Digestion of Bands and Identification of the Peptides and Proteins 

The bands whose intensities were different between the C- and CAF-dams were cut, and an in-gel digestion for mass spectrometric characterization was done, as described by Shevchenko et al. [23]. The different bands detected were excised from the gel with a clean scalpel. Each gel slice was cut into small pieces, and the pieces were transferred to a clean and sterile Eppendorf tube. One hundred microliters ammonium bicarbonate 400 mM and 100 µL of acetonitrile were added and mixed with vortex for 20 min. The supernatant was removed, and the process was repeated. The samples were washed with 100 µL of CH_3_CN, incubated 10 min after wash, the supernatant was removed, and the gel band was dried at room temperature. The samples were kept on ice. The gel band was rehydrated with 20 µL of pre-chilled trypsin solution (20 μg/mL in ammonium bicarbonate 50 mM) and 20 μL of ammonium bicarbonate 50 mM, incubated at 4 °C for 30 min and later at 37 °C overnight. Five µL of 5 % (*v/v*) formic acid was added and mixed by vortex for 5 min. Finally, 30 µL of extraction solution (250 µL of acetonitrile, 25 µL of trifluoroacetic acid and 225 µL of water) was added. The extraction process was repeated with 30 µL of extraction solution. The two extracts were collected together and 1 µL of the extract was deposited on a MALDI-TOF MS plate (polished steel, Bruker–Daltonics) and dried at room temperature. One microliter of matrix (saturated solution α-cyno-4-hydroxy-cinnamic acid in 70/30 acetonitrile/water with 0.1% of trifluoracetic acid) was added and dried at room temperature again. The sample was then analyzed with an Autoflex III MALDI-TOF-TOF (Bruker–Daltonics) spectrometer using the software Compassflex series v1.4 (flexControl v3.4, flexAnalysis v3.4 and BioTools 3.2). MS spectra were recorded in the reflector, positive mode, at a laser frequency of 200 Hz, within a mass range from 500 to 4300 Da. The IS1 voltage was 19 kV, the IS2 voltage was maintained at 16.65 kV, the lens voltage was 8.6 kV, the reflector voltage was 21 kV, and the reflector 2 voltage was 9.6 KV. MS/MS spectra were recorded in the reflector, positive mode, at a laser frequency of 200 Hz. The IS1 voltage was 6 kV, the IS2 voltage was maintained at 5.3 kV, the lens voltage was 3 kV, the reflector voltage 1 was 27 kV, and the reflector 2 voltage was 11.5 KV.

The search process was performed in the Swiss-Prot data base, with the algorithm MASCOT (MatrixSciences), with the following parameters: global modifications: carbamidomethyl (C); variable modification: oxidation (M); mass tol. MS 0.1% and MS/MS tol 0.5 Da.

### 2.6. Quantification of Milk and Plasma Specific Proteins

Milk samples were analyzed for leptin, adiponectin, irisin and haptoglobin concentration, and plasma samples were analyzed for leptin and haptoglobin concentration. Commercial rat ELISA kits were used for the quantification of leptin, adiponectin (R&D Systems, Minneapolis, MN, USA), irisin (Phoenix Pharmaceuticals, Inc., Karlsruhe, Germany) and haptoglobin (Abcam, Cambridge, UK), according to the manufacturer’s instructions. 

### 2.7. Statistical Analyses

No blinding was carried out for data analysis. Data are expressed as the mean ± SEM (*n* = 8). Repeated-measures analysis of variance (ANOVA) followed by a Bonferroni test was used to compare the mean differences between groups in those parameters measured at different time points. In addition, comparisons between two groups were assessed by the non-parametric Mann–Whitney U-test. The test used for each comparison is indicated in the footnote of the figures. The analyses were performed with SPSS for Windows (SPSS, Chicago, IL, USA). Threshold of significance was defined at *p* < 0.05. The Spearman’s correlation coefficient was used to assess the relation between variables, and were performed by the R package (v3.101, R Development Core Team).

## 3. Results

### 3.1. SDS-PAGE Separation, MALDI-TOF MS and MASCOT Protein Identification

**Figure 2:**
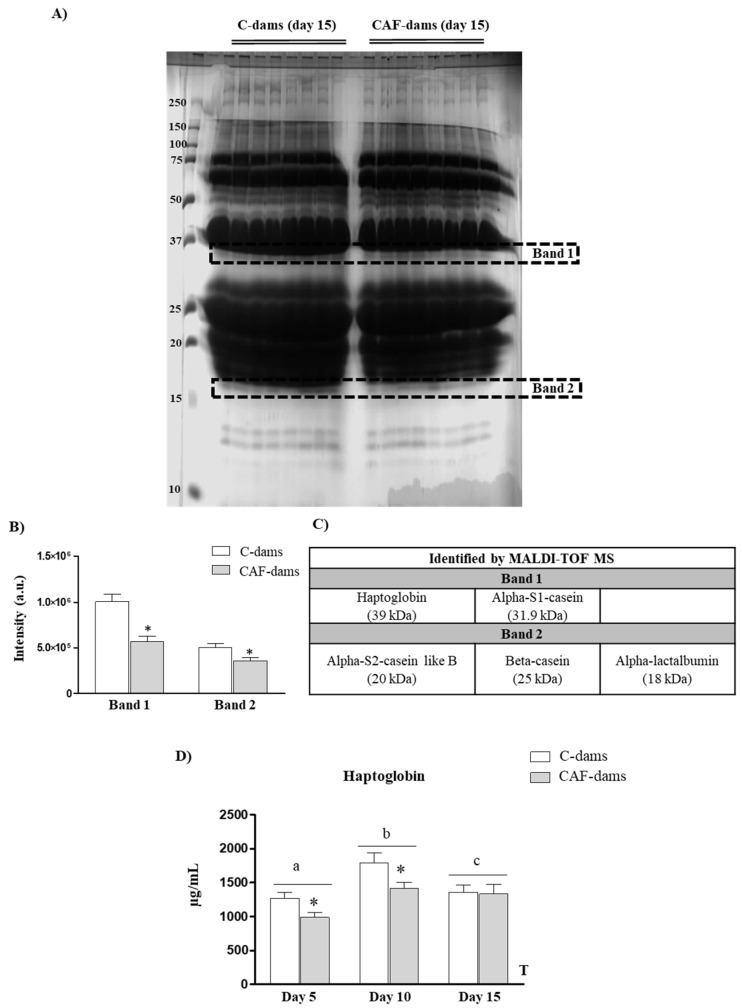
(**A**) Breast milk protein SDS-PAGE (15%) separations stained by Coomassie Blue on day 15 of lactation of control (C-dams) and cafeteria diet-fed dams (CAF-dams). (**B**) Quantification of differential bands and (**C**) identified proteins by peptide mass fingerprinting (matrix-assisted laser desorption/ionization time-of-flight mass spectrometry (MALDI-TOF MS)). (**D**) Concentration of haptoglobin in milk samples at different time points of lactation (day 5, 10 and 15). Data are expressed as the mean ± SEM of 8 dams per group. Statistics: T, effect of time, (*p* < 0.05, ANOVA repeated measures). a ≠ b ≠ c, (*p* < 0.05, Bonferroni post-hoc test). *, cafeteria versus control diet (*p* < 0.05, Mann–Whitney U-test).

Results regarding SDS-PAGE separation are shown in Figure 2A. The intensity of two of the bands, referred to as Band 1 and Band 2, were lower in milk from CAF-dams than in controls (Figure 2B). Subsequent MALDI-TOF MS analysis was performed to identify proteins presents in these bands. Haptoglobin and α-S1-casein were identified in Band 1. Moreover, α-S2-casein like B, β-casein and α-lactalbumin were identified in Band 2. However, we cannot forget that other less abundant proteins, under the limit of detection, were also present.

Levels of haptoglobin in milk samples during lactation (days 5, 10 and 15) were determined by ELISA, to confirm the differences found in the relative between CAF- and C-dams. Haptoglobin concentration in milk was lower in CAF-dams at day 5 and 10, but not at day 15, than in C-dams (Figure 2D). Moreover, haptoglobin concentration in milk changed throughout lactation, peaking on day 10, both in CAF- and C-dams.

### 3.2. Leptin, Adiponectin, Irisin Levels During Lactation 

**Figure 3:**
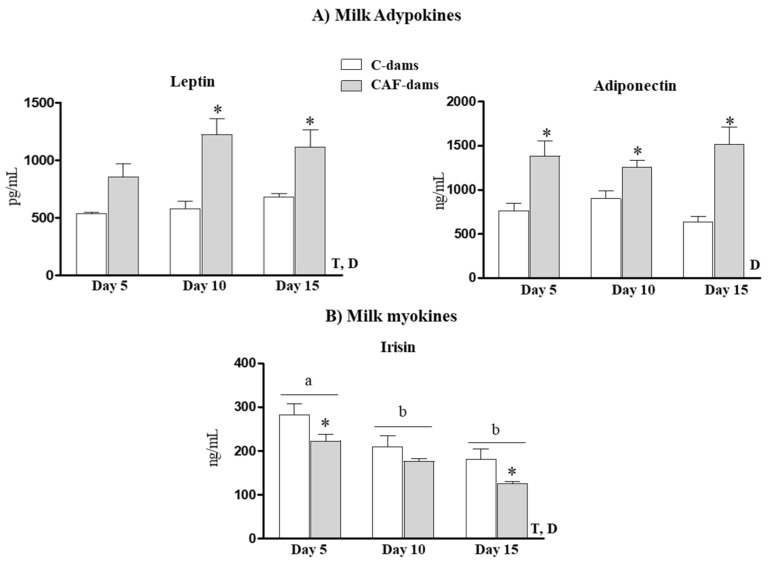
Breast milk leptin, adiponectin and irisin concentration on days 5, 10 and 15 of lactation of control (C-dams) and cafeteria diet-fed dams (CAF-dams). Data are expressed as the mean ± SEM of 8 dams per group. Statistics: D effect of diet (control/cafeteria); T, effect of time, (*p* < 0.05, ANOVA repeated measures). a ≠ b, (*p* < 0.05, Bonferroni post-hoc test). *, cafeteria versus control diet (*p* < 0.05, Mann–Whitney U test).

The levels of selected adipokines (leptin and adiponectin) and the myokine irisin in breast milk on days 5, 10 and 15 of lactation of C- and CAF-dams are presented in Figure 3. CAF-dams displayed greater leptin and adiponectin levels and lower irisin levels than control animals. Milk-borne leptin levels increased throughout the course of lactation, while irisin levels decreased, both in C- and CAF-dams. 

### 3.3. Correlation of Maternal Phenotypic Traits and the Levels of Selected Proteins in Maternal Milk

**Figure 4:**
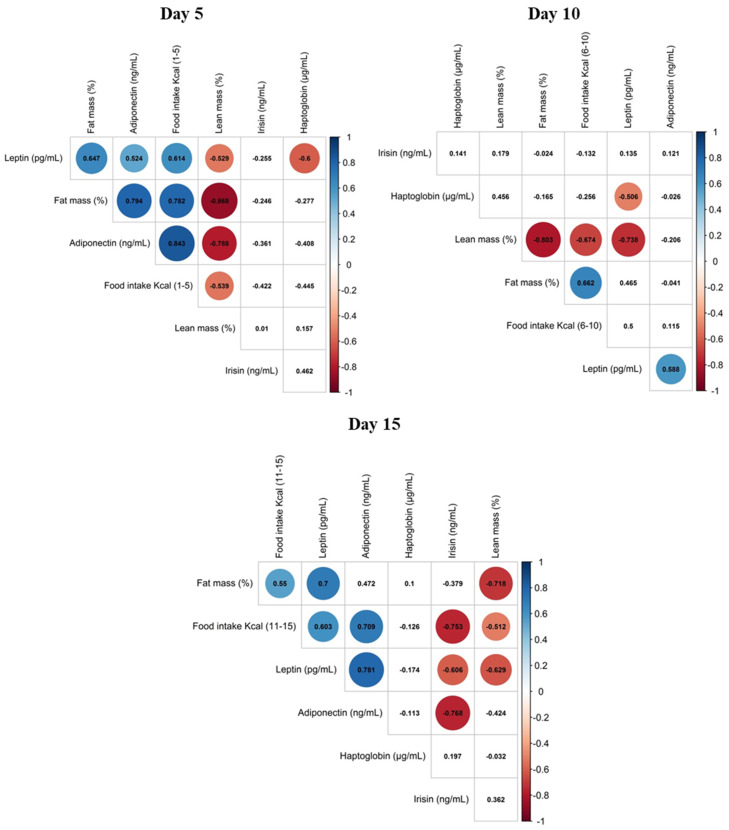
Correlations within maternal food intake, body fat content and lean mass, and leptin, adiponectin, irisin and haptoglobin concentration in milk on days 5, 10 and 15 of lactation of control (C-dams) and cafeteria diet-fed dams (CAF-dams). The comparison was done by non-parametric Rho Spearman correlation. The representation of the correlation matrixes of the Rho values are presented as clusterized heatmaps, in which significant Rho values are replaced by a colored cycle according to the strength of the dependence.

Spearman’s correlation between maternal phenotypic traits (cumulative food intake during the period studied, body fat content and lean mass) and leptin, adiponectin, irisin and haptoglobin proteins levels in milk, on day 5, 10 and 15 are shown in Figure 4. We previously described the phenotypic traits and food intake of C- and CAF-dams [18]. To sum up, CAF-dams had higher body fat content and lower lean mass than controls throughout lactation. In addition, CAF-dams had a higher total energy intake than control, but the absolute intake of proteins was lower [18]. Leptin and adiponectin levels in milk were positively correlated; this correlation was strongest at the end of lactation (PN15). Of note, and as a general trend, milk leptin levels positively correlated with maternal fat mass and negatively correlated with lean mass. At PN5, milk adiponectin levels also correlated with maternal fat mass, but this correlation did not last for the rest of lactating period. Milk levels of both leptin and adiponectin positively correlated with maternal food intake at PN5 and PN15. On the other hand, milk irisin levels were negatively correlated with food intake and milk leptin and milk adiponectin levels at PN15. Moreover, milk haptoglobin levels were negatively correlated with milk leptin levels at PN5 and PN10.

### 3.4. Leptin, Haptoglobin and Irisin Plasma Levels in Offspring During Lactation

**Figure 5:**
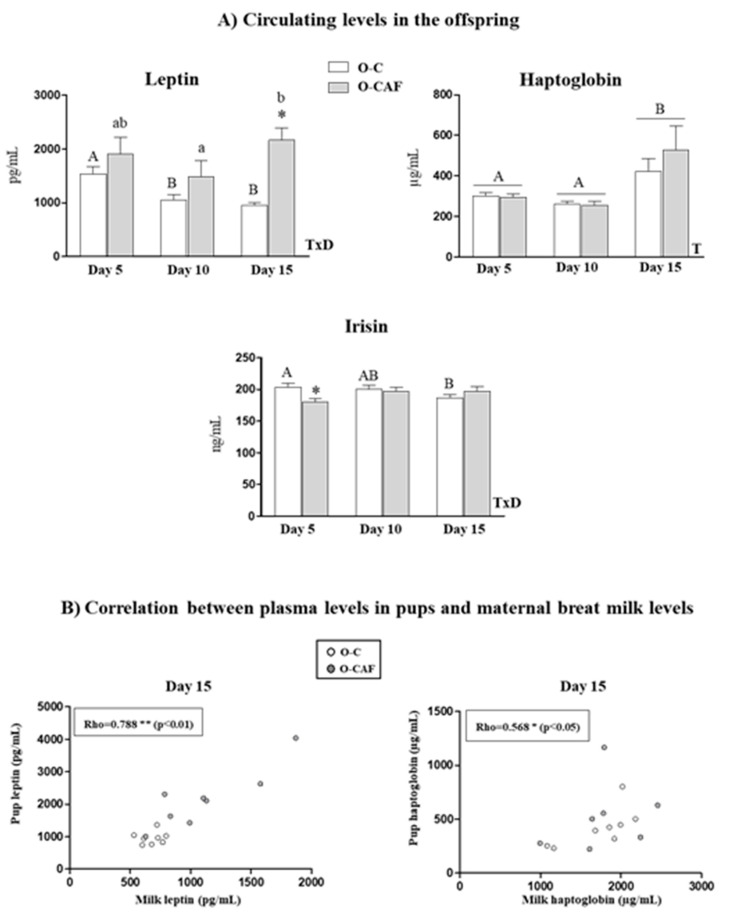
(**A**) Plasma leptin, haptoglobin and irisin concentration on days 5, 10 and 15 of lactation of offspring of dams fed with control (O-C) or a cafeteria diet during lactation (O-CAF). (**B**) Spearman correlations between maternal leptin and haptoglobin concentration in milk and plasma levels in pups on day 15 of lactation of control and cafeteria diet-fed dams. Data are expressed as the mean ± SEM of 16 animals per group. Statistics: T, effect of time; TxD, interactive effect between time and maternal diet, (*p* < 0.05, ANOVA repeated measures). a ≠ b; x ≠ y (*p* < 0.05, Bonferroni post-hoc test). *, cafeteria versus control diet (*p* < 0.05, Mann–Whitney U test). The correlation was done by non-parametric Rho Spearman correlation with a statistical significance (2-tailed) * *p* < 0.05, ** *p* < 0.01.

To assess if changes observed in milk levels may affect circulating levels in the offspring, levels of leptin, haptoglobin and irisin in plasma of suckling pups at PN5, PN10 and PN15 were measured. These results are shown in Figure 5. Regarding leptin levels in pups, the offspring of CAF-dams (O-CAF) displayed greater leptin levels than control pups (O-C) at the end of lactation. A significant positive correlation was found between leptin plasma levels in pups and maternal milk leptin levels (Rho = 0.788, *p* < 0.01), but only at PN15. Despite milk of CAF-dams having lower haptoglobin, no differences were observed in haptoglobin circulating levels between O-CAF and O-C pups. However, haptoglobin levels increased during the suckling period, both in O-C and O-CAF pups. Curiously, at PN15, a positive correlation was found between circulating haptoglobin levels in pups and haptoglobin levels in maternal milk. Regarding irisin levels, O-CAF displayed lower circulating irisin levels on day 5 compared to their controls. However, no correlation with milk irisin levels was found.

## 4. Discussion

A maternal cafeteria diet consumption during lactation was associated with a lower protein content in breast milk [18]. Here, we have screened for those specific proteins whose amount was altered in milk from cafeteria dams. We used SDS-PAGE separation and MALDI-TOF MS approach in milk samples at day PN15 of control and cafeteria diet-fed dams during lactation. 

CAF dams had decreased levels of some caseins (α-S1-casein, α-S2-casein like B, and β-casein), α-lactalbumin and haptoglobin in milk than controls. Casein and α-lactoalbumin in milk are not only a source of amino acids, but also of minerals [2,5]. The lower amount of caseins and α-lactalbumin in milk from CAF-dams may jeopardize a correct absorption of essential minerals and of essential amino acids in the offspring, affecting the functionality and the growth of the offspring. Moreover, studies in adult animals demonstrate that dietary lactalbumin improved energy balance and metabolism, and decreased adiposity [24]. Thus, the lower content of α-lactalbumin in milk of CAF-dams, could contribute to the development of the thin-outside-fat-inside phenotype observed in their offspring previously described in [18]. However, in the present study, we have not directly measured the effects of the lack of α-lactalbumin supplied during lactation; this is a limitation of our study and further research would be needed to stablish the cause-effect of α-lactalbumin supplied by maternal milk.

Milk from CAF-dams had a lower content of haptoglobin. We have confirmed MALDI-TOF MS results by ELISA. We also found decreased levels of haptoglobin on day 5 and 10 of lactation in the milk of the cafeteria dams. However, there were no significant differences in haptoglobin levels on day 15 of lactation. The structure of haptoglobin is similar to immunoglobulin. It consists of two heavy β-chains and two light chains: α1 and α2 [25]. Its most widely characterized function is to bind to cell-free haemoglobin for degradation in the liver, and for iron recycling [26]. Originally, haptoglobin was isolated in the liver as part of the acute phase response to inflammation, thus, haptoglobin has long considered a marker of inflammation (reviewed in [27]). In addition, levels of haptoglobin correlate positively with body mass index, therefore it is considered a marker of adiposity (reviewed in [27]). The adipose tissue is also an important source for haptoglobin, contributing to its circulating levels [28]. In fact, only after 5 days with a HF diet, mice showed up-regulated mRNA levels of haptoglobin in white adipose tissue (WAT) [29]. In breast milk, haptoglobin levels have been proposed as a biomarker of subclinical mastitis in dairy cows [30]. Here, we do not observe any negative effect of cafeteria feeding on haptoglobin levels in milk; in fact, CAF-dams present a lower concentration of haptoglobin in milk than control dams. The understanding of the biological significance of haptoglobin within the breast milk related to the development of suckling pups is not known. In addition, strong evidences of their bioavailability (if any) in suckling pups are lack. In this sense, the decrease of haptoglobin in milk from CAF- dams are not reflected in circulating haptoglobin levels in the offspring. The higher haptoglobin levels found in cafeteria pups may not be directly related to the levels supplied by maternal milk, but could reflect endogenous production by the WAT and the greater adiposity of the pups. Indeed, haptoglobin mRNA was up-regulated in WAT of many murine obese models, such as genetically obese ob/ob, and dietary obese model obtained by a high-fat (HF) diet [29,31].

We previously described that CAF-dams had a greater cumulative food intake and adiposity than controls during whole lactation [18]. Interestingly, leptin and adiponectin levels in milk correlate positively with maternal cumulative food intake and fat mass, particularly leptin, almost in all the studied time points of lactation. Previous studies also have reported that breast milk leptin concentrations were significantly correlated with mothers’ body mass index (BMI) [8,9]. For example, overweight and obese mothers displayed higher milk leptin levels than normal weight mothers [32]. In addition, observational studies in humans had shown that maternal milk leptin levels negatively correlate with BMI increases of the infant [8,33,34]. 

CAF-dams presented greater levels of leptin in breast milk than controls. Previous studies in animals models from our group provided evidence that exogenous leptin supplementation to neonate rats has beneficial effects and prevents body weight gain in adulthood, improves the metabolic response of adipose tissue to a HF diet, and improves insulin sensitivity in adulthood (reviewed in [35]). However, the offspring of cafeteria diet-fed dams, despite the higher maternal milk leptin supply during lactation, presented greater fat mass at weaning and later on in the adulthood than their controls [18]. It is worth noting that the above-mentioned animal studies of the beneficial role of leptin supplementation during lactation, was done in pups whose mothers were under a control diet. Here, the offspring of cafeteria pups presented also greater plasma leptin levels, reflecting the higher leptin intake throughout breast milk. However, it cannot be ruled out the contribution of others’ bioactive components of milk that increases the endogenous production of leptin in pups. Thus, the maternal intake of a cafeteria diet, or a specific component of this diet, may counteract or block the expected beneficial effect for the higher leptin supply during lactation. Therefore, more studies are needed, dealing with the antagonist effects of maternal diet in structural, post-transcriptional modification or the mechanism action of maternal milk leptin. Interestingly, leptin is a milk glycosylated protein [36]. Protein glycosylation is one of the most commonly occurring in post-translational modifications, and directly affecting protein structure and function [37,38]. While milk proteins have been studied for decades, strikingly little effort has been applied to determining differences in post-translational modifications. Glycoproteins and their digestive products provide an important source of bioactive compounds, with diverse beneficial properties [37]. Therefore, it seems interesting not only to study the amounts of specifics proteins in milk, but also the changes in its glycosylation status, which could play important roles in the ligand–receptor interactions in the offspring. Here, we have not checked the glycosylation state of milk leptin, or if there were any differences in the glycosylation state due to maternal cafeteria diet consumption. Thus, we cannot conclude that the post-transcriptional modification in leptin supplied in milk may affect their beneficial properties.

Regarding adiponectin, CAF-dams presented greater levels of adiponectin in breast milk than controls. In human milk, the concentration of adiponectin is more than 20–100 times that of other major adipokines of milk such as leptin [39]. Adiponectin plasma levels decreased in metabolic disorders, such as type 2 diabetes, insulin resistance and dyslipidaemia. Therefore, adiponectin plasma levels exert insulin sensitizing and anti-inflammatory effects [40]. Despite the inverse relationship between circulating adiponectin levels and adiposity, as higher maternal adiposity, more adiponectin levels in human milk are found [10]. These have raised questions regarding its biological relevance [41]. Moreover, adiponectin levels in breast milk have been associated with greater weight gain and higher fat mass in the offspring at the age of 2 years [14,42]. Thus, milk adiponectin has been suggested as a factor risk for childhood overweight [14]. In animal models, it has been described that both maternal adiponectin overexpression and deletion in mice lead to systemic inflammation in sucking pups [43]. The lack of adiponectin is related to the increase in proinflammatory cytokines in the mammary gland, while adiponectin overabundance results in excessive long-chain saturated fatty acids in milk [43]. 

Irisin is also present in milk [11]. Colostrum had greater irisin concertation than transitional milk, and mature milk had the lowest concentrations of irisin [11]. Moreover, lactating women with gestational diabetes mellitus presented lower irisin concentrations in the colostrum, transitional and mature milk than healthy lactating women [11]. Information regarding irisin levels and their putative role are scarce. Results obtained in our laboratory using another and independent experimental design, showed that milk irisin levels decreased thought lactation in rats [44]. In the present study, we also show a decrease in milk irisin levels during lactation. In addition, CAF-dams displayed lower milk irisin levels than controls. However, any correlation with maternal biometrical parameters was observed, and thus, we cannot establish any relationship between milk irisin and maternal adiposity. In addition, O-CAF displayed lower irisin levels in pups at day 5 compared to controls, in accordance to the lower levels in maternal milk, suggesting that milk born irisin could contribute in irisin circulating levels in pups, at least at the first stage of lactation. In addition, we cannot discard the effect of other bioactive components altered in milk of cafeteria fed dams could deregulate endogenous synthesis of irisin.

## 5. Conclusions

In conclusion, maternal intake of an unbalanced diet, such as a cafeteria diet, influences the concentration of specific proteins in milk (such as leptin, adiponectin, irisin and haptoglobin). Further studies need to be done to address the specific contribution to each peptide or the possible synergies among other milk bioactive components. However, considering that proteins may exert a biological role beyond their nutritional role as a source of amino acids, they could affect the metabolic health of the offspring. Although no direct cause-effects can be extrapolated from this study, if these changes imply alterations in protein levels with a regulatory function in the offspring, these should be considered, to assure a correct nutrition during an important developmental phase.
